# Association between smoking and anemia in adult women in Peru: analysis of a national survey (ENDES) 2023

**DOI:** 10.1186/s12889-025-23976-7

**Published:** 2025-08-19

**Authors:** Karina I. Torrejon-Echaccaya, Nelson J. Mathews-Haro, David Rodriguez-Simbron, David R. Soriano-Moreno

**Affiliations:** School of Medicine, Universidad Peruana Unión, Carretera Central Km 19.5 Ñaña, Chosica, Lima, Peru

**Keywords:** Anemia, Tobacco use disorder, Peru

## Abstract

**Background:**

Anemia is a major public health issue that disproportionately affects women of reproductive age. While tobacco use may influence hemoglobin levels through physiological mechanisms, its association with anemia remains unclear. This study aimed to evaluate the relationship between smoking and anemia in adult women in Peru using nationally representative data.

**Methods:**

Cross-sectional study based on secondary data from a national survey (ENDES 2023). Women aged 18 to 49 years with complete information on the variables of interest were included. The independent variable was smoking, and the dependent variable was the anemia status. To evaluate the association between variables, Poisson regression with robust variance was used to calculate prevalence ratios (PR).

**Results:**

A total of 13 036 women with a mean age of 33.0 years were analyzed. No significant association was found between cigarette smoking in the last 12 months (aPR: 1.01; 95% CI: 0.82 to 1.24), smoking in the last 30 days (aPR: 0.89; 95% CI: 0.66 to 1.22), or daily smoking (aPR: 1.08; 95% CI: 0.55 to 2.14) and the prevalence of anemia. On the other hand, a higher prevalence of anemia was observed in women aged 30 to 49 years. While the factors associated with a lower prevalence of anemia were black ethnicity, overweight, obesity, and living in small cities.

**Conclusion:**

No significant association was found between smoking and the presence of anemia in Peruvian adult women. However, factors such as age, nutritional status and ethnicity were associated with the prevalence of anemia.

## Introduction

Anemia remains a global public health issue, with a particularly significant impact on women of reproductive age. According to the World Health Organization, approximately 29.9% of women worldwide are affected by this condition [1]. The burden is even higher in low- and middle-income countries, where it affects 39.5% of women [2]. In Peru, data from the 2023 Demographic and Health Survey (ENDES) report a prevalence of 22.7% among women aged 15 to 49 years [3]. This condition is associated with multiple adverse outcomes, including reduced physical performance, difficulty concentrating, impaired quality of life, and, in pregnant women, increased risk of maternal and neonatal morbidity and mortality [4].

The etiology of anemia is multifactorial and includes nutritional causes, chronic and infectious diseases, and socioeconomic conditions. However, a modifiable and less studied factor is smoking. Tobacco use could negatively influence hematological status through multiple pathophysiological mechanisms such as interference in the absorption and metabolism of essential micronutrients such as iron, vitamin B12 and folic acid; the induction of oxidative stress and hemolysis; as well as the increased risk of chronic diseases [5]. On the other hand, it has been proposed that smoking could induce chronic hypoxia that stimulates the production of erythropoietin, which would result in higher levels of hemoglobin [6].

Previous studies that have evaluated this association have reported conflicting results. A case-control study in India found a strong association between smoking and iron deficiency anemia, particularly among light and long-term smokers. Also, research in Ghana linked both active and passive smoke exposure to lower hemoglobin levels and increased anemia risk [7, 8]. Conversely, studies in Bosnia and the United States reported higher hemoglobin concentrations among smokers [9, 10]. Additionally, emerging evidence suggests that exposure to other types of air pollutants may also be associated with anemia, further supporting the plausibility of this relationship [11, 12]. These inconsistencies may stem from differences in population characteristics, measurement approaches, and exposure levels. Moreover, existing studies have certain limitations such as non-representative samples, or absence of sex-stratified data, especially in women of reproductive age who are at increased risk of anemia.

In Peru, the health burden of tobacco remains significant, contributing to the loss of over 515,000 years of life annually and accounting for 1.28% of the gross domestic product [13]. These figures underscore the importance of evaluating the broader health consequences of tobacco use, including its potential association with anemia. In addition, this relationship has not been addressed in Latin American contexts, where sociodemographic, cultural, and access to the health system characteristics could modify this association. Understanding this relationship among Peruvian adult women is crucial for the development of anemia prevention policies where tobacco control could be integrated into their strategies.

Therefore, the objective of this study was to evaluate the association between smoking and anemia in adult women in Peru.

## Materials and methods

### Design and population

This research has a cross-sectional analytical design based on a secondary analysis of ENDES 2023. The ENDES is an annual national survey representative of the Peruvian population prepared by the National Institute of Statistics and Informatics (INEI) that has a probabilistic, multistage sampling design. Female participants aged 18 to 49 years, regardless the smoking status, with complete data on the variables of interest were included.

### Variables

The dependent variable was anemia found in the database as “Anemia Level” (HA57) and includes the categories of severe anemia (< 7 g/dl), moderate anemia (7.0 to 9.9 g/dl), mild anemia (10 to 11.9 g/dl (10 to 10.9 for children and pregnant women)) and no anemia. This variable was recategorized into present (mild to severe) and absent anemia. Hemoglobin levels were adjusted for altitude.

The independent variable was smoking measured with the variable “In the last 12 months you have smoked cigarettes” (QS200), “In the last 30 days you have smoked cigarettes” (QS201), and “You smoke daily” (QS202).

Other covariates were age (years), place of residence (capital or large city, small city, town, countryside), educational level (no education, primary, secondary, higher education), marital status (single, married, cohabiting, divorced, separated, widowed), wealth index (poorest, poor, middle, rich, the richest), ethnicity (mestizo, Quechua, Aymara, black, white, other, don’t know/don’t answer), having health insurance (no, yes), body mass index (normal weight, underweight, overweight, obesity), current pregnancy (no/I do not know, yes), and if you have ever consumed alcoholic beverages (no, yes).

### Statistical analysis

The data used were publicly available on the INEI website [14]. Statistical analyses were conducted using STATA version 17.0. The survey databases were merged using unique identification keys. All analyses accounted for the complex survey design by applying the svy command. Participants with missing data for the variables of interest were excluded from the analysis. The statistical power to detect a minimum difference of 5% in anemia prevalence was 93.9% for smoking in the last 12 months, 71.2% for smoking in the last 30 days, and 22.0% for daily smoking. Categorical variables were presented as proportions with their corresponding 95% confidence intervals (95% CI). Numerical variables were presented as means with 95% CI, assuming a normal distribution. To assess the association between smoking and anemia, we used Poisson regression models with robust variance to estimate crude prevalence ratios (PRc) and adjusted prevalence ratios (aPR), along with their 95% CIs. All covariates were included in the adjusted model based on epidemiological relevance, except for “Education level,” “Health insurance status,” and “Alcohol consumption history,” which were excluded due to multicollinearity (variance inflation factor > 10). Age was categorized for the same reason. A p-value < 0.05 was considered statistically significant.

### Ethical considerations

The study protocol was approved by the Ethics Committee of the Universidad Peruana Unión (approval code: 2025-CEB-FCS - UPeU-° 0031). This study followed the ethical principles of the Declaration of Helsinki. All participants provided oral informed consent to take part in the survey and for hemoglobin measurement. The analysis used publicly available and anonymized secondary data from the ENDES 2023 survey. 

## Results

**Fig. 1:**
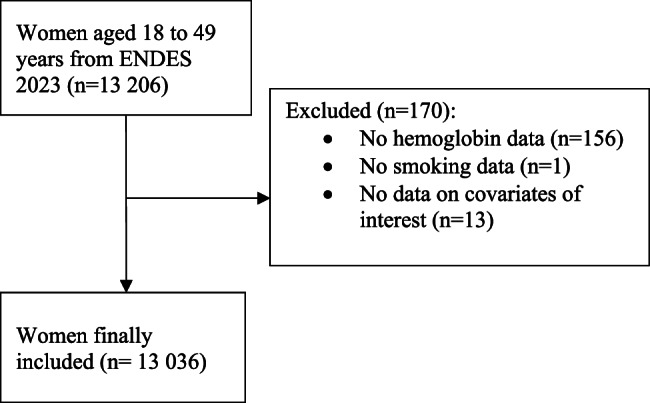
Participant inclusion flowchart

Initially, 13 206 women aged 18 to 49 years were found in the ENDES database. Due to missing data in the variables of interest, 170 participants were excluded. Finally, we included 13 036 women for the present study (Fig. 1).

Regarding sociodemographic characteristics, the mean age of the participants was 33.0 years. Most lived in a large city (36.2%), had completed secondary education (45.0%), were cohabiting (45.7%), belonging to the poor wealth index category (21.8%), identified as mestizo (48.1%), and reported having health insurance (89.0%). Additionally, the majority were overweight (38.8%) and reported not being currently pregnant (97.8%). Concerning harmful behaviors, 93.3% reported having ever consumed alcoholic beverages, while 7.7% (95% CI: 6.9 to 8.6%) had smoked cigarettes in the last 12 months, 3.7% (95% CI: 3.2 to 4.3%) in the last 30 days, and 0.7% (95% CI: 0.5 to 1.1%) reported daily smoking. The mean hemoglobin level was 126.4 g/L, and 23.0% (95% CI: 21.8 to 24.2%) of participants had some degree of anemia, with mild anemia being the most frequent (19.9%) (Table 1).

**Table 1:** Characteristics of the participants (*n* = 13 036)

Variables		%(95% CI)
Age, mean		33.0(32.7 to 33.2)
Place of residence		
Capital or large city		36.2(34.7 to 37.7)
Small city		22.5 21.6 to 23.4)
Town		24.0(23.1 to 24.9)
Countryside(rural area)		17.3(16.6 to 18.0)
Educational level		
No education		1.1(0.8 to 1.3)
Primary		13.6(12.8 to 14.4)
Secondary		45.0(43.7 to 46.4)
Higher		40.3(39.0 to 41.7)
Marital status		
Single		16.3(15.1 to 17.5)
Married		18.6(17.6 to 19.7)
Cohabiting		45.7(44.4 to 47.1)
Divorced, Separated, or Widowed		19.3(18.2 to 20.5)
Wealth Index		
The poorest		18.1(17.3 to 18.9)
Poor		21.8(20.6 to 22.9)
Middle		21.5(20.4 to 22.7)
Rich		21.6(20.3 to 22.9)
The richest		17.0(15.8 to 18.2)
Ethnicity		
Mestizo		48.1(46.7 to 49.6)
Quechua		23.2(22.1 to 24.4)
Aymara		1.8(1.5 to 2.1)
Black		11.3(10.5 to 12.1)
White		8.0(7.2 to 8.8)
Other		2.5(2.1 to 2.9)
Don’t know/Don’t answer		5.2 (4.5 to 5.9)
Having health insurance		
No		11.0 (10.1 to 12.1)
Yes		89.0 (87.9 to 89.9)
Body Mass Index		
Normal weight		30.0 (28.8 to 31.3)
Underweight		0.8 (0.6 to 1.1)
Overweight		38.8 (37.5 to 40.1)
Obesity		30.4 (29.1 to 31.7)
Currently pregnant		
No/I do not know		97.8 (97.4 to 98.2)
Yes		2.2 (1.8 to 2.6)
Have ever consumed alcoholic beverages		
No		6.7 (6.1 to 7.3)
Yes		93.3 (92.7 to 93.9)
Smoked in the last 12 months		
No		92.3 (91.4 to 93.0)
Yes		7.7 (6.9 to 8.6)
Smoked in the last 30 days		
No		96.3 (95.7 to 96.8)
Yes		3.7 (3.2 to 4.3)
Smoke daily		
No		99.3 (98.9 to 99.5)
Yes		0.7 (0.5 to 1.1)
Hemoglobin (g/L) adjusted for altitude, mean		126.4 (126.0 to 126.7)
Level of anemia		
No anemia		77.0 (75.8 to 78.2)
Mild		19.9 (18.7 to 21.0)
Moderate		2.8 (2.4 to 3.4)
Severe		0.3 (0.2 to 0.5)
Anemia		
No anemia		77.0 (75.8 to 78.2)
With anemia		23.0 (21.8 to 24.2)

95% confidence interval

95% CI

The prevalence of anemia was significantly higher among adult participants aged 30 to 49 years (24.3%, *p* = 0.007), those of Aymara or other ethnicities (27.1% and 29.3%, respectively; *p* = 0.003), and those with underweight (34.5%, *p* < 0.001). In contrast, the prevalence of anemia was similar among participants who did not smoke and those who reported smoking cigarettes in the last 12 months, the last 30 days, or daily (Table 2).

**Table 2:** Characteristics of the participants according to anemia (*n* = 13 036)

Variables		Anemia		*P*value*
		Absent (77.0%)	Present (23.0%)	
		% (95% CI)	% (95% CI)	
Age, mean		32.7 (32.4 to 33.1)	33.7 (33.1 to 34.3)	< 0.001
Age categorized				
Young adult (18 to 29 years old)		79.0 (77.2 to 80.8)	20.9 (19.2 to 22.8)	0.007
Adult (30 to 49 years)		75.7 (74.1 to 77.3)	24.3 (22.7 to 25.9)	
Place of residence				
Capital or large city		75.2 (72.3 to 78.0)	24.8 (22.0 to 27.7)	0.09
Small city		78.7 (77.0 to 80.4)	21.3 (19.6 to 23.0)	
Town		77.8 (76.0 to 79.5)	22.2 (20.5 to 24.0)	
Countryside (rural area)		77.5 (75.6 to 79.3)	22.5 (20.8 to 24.4)	
Educational level				
No education		66.9 (55.9 to 76.3)	33.1 (23.7 to 44.0)	0.16
Primary		78.6 (76.1 to 80.9)	21.4 (19.1 to 23.9)	
Secondary		77.3 (75.4 to 78.9)	22.7 (21.0 to 24.6)	
Higher		76.5 (74.3 to 78.5)	23.5 (21.5 to 25.7)	
Marital status				
Single		77.7 (74.1 to 81.0)	22.3 (19.0 to 25.9)	0.489
Married		75.0 (71.8 to 77.9)	25.0 (22.1 to 28.2)	
Cohabiting		77.6 (75.9 to 79.2)	22.4 (20.8 to 24.1)	
Divorced, Separated, or Widowed		76.9 (74.2 to 79.5)	23.1 (20.5 to 25.8)	
Wealth Index				
The poorest		77.3 (75.4 to 79.1)	22.7 (20.9 to 24.6)	0.242
Poor		78.3 (76.0 to 80.3)	21.8 (19.7 to 24.0)	
Middle		78.0 (75.3 to 80.5)	22.0 (19.5 to 24.7)	
Rich		77.0 (73.9 to 79.7)	23.0 (20.3 to 26.0)	
The richest		73.9 (69.9 to 77.7)	26.0 (22.3 to 30.1)	
Ethnic group				
Mestizo		76.1 (74.1 to 77.9)	23.9 (22.1 to 25.9)	0.003
Quechua		75.9 (73.5 to 78.0)	24.1 (22.0 to 26.5)	
Aymara		72.9 (66.2 to 78.7)	27.1 (21.3 to 33.8)	
Black		82.7 (79.7 to 85.4)	17.3 (14.6 to 20.3)	
White		78.6 (73.5 to 82.9)	21.5 (17.1 to 26.5)	
Other		70.7 (64.2 to 76.4)	29.3 (23.6 to 35.8)	
Don’t know/Don’t answer		80.8 (75.0 to 85.4)	19.2 (14.6 to 24.9)	
Having health insurance				
No		77.1 (72.9 to 80.9)	22.9 (19.1 to 27.1)	0.955
Yes		77.0 (75.7 to 78.3)	23.0 (21.7 to 24.3)	
Body Mass Index				
Normal weight		73.3 (71.0 to 75.5)	26.7 (24.5 to 29.0)	< 0.001
Underweight		65.5 (50.8 to 77.7)	34.5 (22.3 to 49.3)	
Overweight		78.4 (76.5 to 80.2)	21.6 (19.8 to 23.5)	
Obesity		79.2 (76.9 to 81.4)	20.8 (18.6 to 23.1)	
Currently pregnant				
No/I do not know		76.9 (75.7 to 78.2)	23.0 (21.8 to 24.3)	0.526
Yes		78.9 (72.5 to 84.3)	21.0 (15.8 to 27.5)	
Have ever consumed alcoholic beverages				
No		77.2 (72.9 to 80.9)	22.8 (19.1 to 27.0)	0.929
Yes		77.0 (75.7 to 78.3)	22.9 (21.7 to 24.3)	
Smoked in the last 12 months				
No		77.0 (75.8 to 78.2)	22.9 (21.8 to 24.2)	0.991
Yes		77.0 (71.9 to 81.4)	23.0 (18.6 to 28.1)	
Smoked in the last 30 days				
No		76.9 (75.7 to 78.1)	23.1 (21.9 to 24.3)	0.474
Yes		79.4 (72.3 to 85.0)	20.6 (15.0 to 27.7)	
Smoke daily				
No		77.0 (75.8 to 78.2)	23.0 (21.8 to 24.2)	0.826
Yes		75.2 (55.4 to 88.1)	24.8 (11.9 to 44.6)	

*Pvalue calculated with chi2 or Student’s T

In the regression analysis, no significant association was found between cigarette smoking in the last 12 months (aPR: 1.01; 95% CI: 0.82 to 1.24), having smoked in the last 30 days (aPR: 0.89; 95% CI: 0.66 to 1.22), or daily smoking (aPR: 1.08; 95% CI: 0.55 to 2.14) and the prevalence of anemia. On the other hand, an age between 30 and 49 years was associated with a higher prevalence of anemia (aPR: 1.21; 95% CI: 1.07 to 1.35). While the factors associated with a lower prevalence of anemia were living in a small city (aPR: 0.86; 95% CI: 0.74 to 0.98), be of black ethnicity (aPR: 0.76; 95% CI: 0.63 to 0.92), overweight (aPR: 0.78; 95% CI: 0.69 to 0.88) and obesity (aPR: 0.74; 95% CI: 0.64 to 0.85) (Table 3).

**Table 3:** Association between smoking and anemia in women in Peru (*n* = 13 036)

Variable		Crude PR (95% CI)	Adjusted PR (95% CI)*
Smoked in the last 12 months			
No		Ref.	Ref.
Yes		1.00 (0.81 to 1.24)	1.01 (0.82 to 1.24)
Smoked in the last 30 days			
No		Ref.	Ref.
Yes		0.89 (0.66 to 1.22)	0.89 (0.66 to 1.22)
Smoke daily			
No		Ref.	Ref.
Yes		1.08 (0.55 to 2.12)	1.08 (0.55 to 2.14)
Age categorized			
Young adult (18 to 29 years old)		Ref.	Ref.
Adult (30 to 49 years)		1.16 (1.04 to 1.29)	1.21 (1.07 to 1.35)
Place of residence			
Capital or large city		Ref.	Ref.
Small city		0.86 (0.75 to 0.99)	0.86 (0.74 to 0.98)
Town		0.90 (0.78 to 1.03)	0.92 (0.80 to 1.06)
Countryside (rural area)		0.91 (0.79 to 1.05)	0.90 (0.76 to 1.08)
Educational level			
No education		Ref.	Not included
Primary		0.65 (0.47 to 0.90)	
Secondary		0.69 (0.50 to 0.95)	
Higher		0.71 (0.51 to 0.98)	
Marital status			
Single		Ref.	Ref.
Married		1.12 (0.93 to 1.36)	1.09 (0.89 to 1.34)
Cohabiting		1.00 (0.85 to 1.19)	1.04 (0.88 to 1.24)
Divorced, Separated, or Widowed		1.03 (0.86 to 1.25)	1.05 (0.86 to 1.27)
Wealth Index			
The poorest		Ref.	Ref.
Poor		0.96 (0.84 to 1.09)	0.97 (0.84 to 1.13)
Middle		0.97 (0.84 to 1.12)	0.97 (0.82 to 1.16)
Rich		1.01 (0.87 to 1.18)	0.97 (0.81 to 1.17)
The richest		1.15 (0.96 to 1.36)	1.08 (0.87 to 1.34)
Ethnic group			
Mestizo		Ref.	Ref.
Quechua		1.01 (0.89 to 1.14)	1.02 (0.90 to 1.16)
Aymara		1.13 (0.88 to 1.45)	1.20 (0.94 to 1.54)
Black		0.72 (0.60 to 0.87)	0.76 (0.63 to 0.92)
White		0.90 (0.71 to 1.13)	0.91 (0.72 to 1.14)
Other		1.22 (0.98 to 1.53)	1.25 (1.00 to 1.56)
Don’t know/Don’t answer		0.80 (0.61 to 1.07)	0.81 (0.61 to 1.07)
Having health insurance			
No		Ref.	Not included
Yes		1.01 (0.84 to 1.21)	
Body Mass Index			
Normal weight		Ref.	Ref.
Underweight		1.29 (0.86 to 1.95)	1.34 (0.90 to 2.01)
Overweight		0.81 (0.72 to 0.91)	0.78 (0.69 to 0.88)
Obesity		0.78 (0.68 to 0.89)	0.74 (0.64 to 0.85)
Currently pregnant			
No/I do not know		Ref.	Ref.
Yes		0.91 (0.69 to 1.21)	0.98 (0.74 to 1.31)
Have ever consumed alcoholic beverages			
No		Ref.	Not included
Yes		1.01 (0.84 to 1.21)	
*PR P*revalence ratio, 95% *CI* 95% Confidence interval, *Ref* Reference			

* Smoking in the last 12 months, last 30 days, and daily smoking were adjusted for age, place of residence, marital status, wealth index, ethnic group, body mass index, and current pregnancy status. Educational level, health insurance coverage, and history of alcohol consumption were excluded from the model due to multicollinearity. Estimates for the other covariates are derived from the model that included smoking in the last 12 months.

## Discussion

The present cross-sectional study, based on nationally representative data, did not find a significant association between smoking and the presence of anemia. However, it was identified that the age of 30 to 49 years was associated with a higher prevalence of anemia, while the factors associated with a lower prevalence of anemia were black ethnicity, overweight, obesity, and living in small cities.

The absence of a significant association between smoking and anemia coincides with the heterogeneity reported in the literature. A previous case-control study conducted in India found that tobacco smoking was strongly associated with anemia (OR: 7.72, *p* < 0.001) [7]. Likewise, another cross-sectional study conducted in Ghana found that hemoglobin levels were significantly lower in those who smoked almost daily (β: −1.40; 95% CI: −2.01 to −0.79), at least once a month (β: −1.14; 95% CI: −1.79 to −0.48) or were exposed to secondhand smoke (β: −0.77; 95% CI: −1.30 to −0.21) [8]. Similarly, another study conducted in an older adult population in India found that the odds of anemia were higher in those with a history of tobacco abuse (aOR: 1.23; 95% CI: 1.12 to 1.35) [11]. On the other hand, a study conducted in Bosnia and Herzegovina revealed that smokers had significantly higher levels of hemoglobin (14.7 vs. 13.9 g/dL, *p* = 0.042) [9]. Finally, a study conducted in the United States found that in both women and men, smokers had significantly higher average hemoglobin levels than non-smokers [10].

From a pathophysiological point of view, these differences could be due to the fact that smoking can decrease hemoglobin levels due to mechanisms such as interference with iron and vitamin absorption, oxidative stress, and increased concurrent chronic diseases [5]. In contrast, it has been proposed that smoking may raise hemoglobin levels due to chronic hypoxia induced by carboxyhemoglobin formation following exposure to carbon monoxide from cigarette smoke, resulting in a compensatory response that is not necessarily beneficial, as it may favor a state of hypercoagulability [15, 16].

In addition, differences in the characteristics of the populations studied, levels of exposure to tobacco, the type of products smoked, exposure to other types of biomasses, nutritional status and the presence of underlying inflammatory or infectious conditions must be considered. Also, it is important to take into account that the results were consistent between smoking the last 12 months, the last 30 days and smoking daily, which reinforces the robustness of our conclusions.

Regarding other variables associated with anemia, it was observed that women between 30 and 49 years of age had a higher prevalence of anemia compared to younger women, a finding that is consistent with previous studies [17]. On the other hand, black ethnicity was associated with a lower prevalence of anemia, a finding discordant with previous literature [18], probably because there are contextual factors specific to Peru, such as differences in access to health services, dietary patterns, or biases in self-reported ethnic classification, which could have influenced this result. Another interesting finding was that overweight and obesity were associated with lower prevalence of anemia, which coincides with previous studies suggesting that excess weight may be related to higher iron and vitamin C intake [19].

### Implications and recommendations

These findings provide relevant local evidence that could guide future anemia prevention strategies in adult women. Although no association was found, smoking remains a leading modifiable risk factor for multiple chronic conditions and continues to impose a high economic and disease burden in Peru. As highlighted by national estimates, smoking is responsible for over 22,000 deaths and 126,000 disease events annually, with a substantial loss in productivity and health system costs [13]. Therefore, tobacco control policies should be sustained and strengthened, not only for their established benefits in reducing cardiovascular, respiratory, and cancer outcomes, but also to ensure comprehensive health protection. Importantly, these findings are specific to women and should not be extrapolated to men, where tobacco use is more prevalent and the potential impact on hematological outcomes may differ [20]. In addition, the findings highlight the importance of considering nutritional status, ethnicity, and age when designing interventions to reduce the burden of anemia in women of reproductive age.

### Future research directions

It is recommended that future studies be longitudinal to better understand how smoking influences anemia risk. Moreover, it would be relevant to evaluate the intensity, duration and type of tobacco consumption. Also, future research should explore how anemia is affected by secondhand smoke exposure, particularly in household and occupational settings, as well as exposure to indoor air pollution from biomass fuels such as wood or charcoal, which remain common sources of energy in many regions of Peru.

### Limitations and strengths

The present study has several limitations. Firstly, the cross-sectional design prevents establishing temporality. Analyses could not be performed according to the intensity, duration and type of tobacco use. However, in this study we used a national database representative of the Peruvian population that includes the main sociodemographic and clinical covariates to perform an appropriate adjusted analysis.

## Conclusion

In the present study, no significant association was found between smoking and the presence of anemia in Peruvian adult women. Other factors associated with the prevalence of anemia were age, nutritional status, and ethnicity. These findings highlight the need for comprehensive policies that consider these factors in the prevention of anemia.

## Data Availability

The datasets analyzed during the current study are publicly available from the INEI website.
